# Development and In Vitro and In Vivo Evaluation of Microspheres Containing Sodium *N*-[8-(2-hydroxybenzoyl)amino]caprylate for the Oral Delivery of Berberine Hydrochloride

**DOI:** 10.3390/molecules25081957

**Published:** 2020-04-23

**Authors:** Ying Li, Chunyan Zhu

**Affiliations:** Department of Pharmaceutics, Institute of Medicinal Plant Development, Chinese Academy of Medical Sciences and Peking Union Medical College, No. 151 Malianwa North Road, Haidian District, Beijing 100193, China; lysole@126.com

**Keywords:** sodium *N*-[8-(2-hydroxybenzoyl)amino]caprylate, permeation enhancement, berberine hydrochloride, microspheres, oral bioavailability

## Abstract

Microspheres containing absorption enhancer (sodium *N*-[8-(2-hydroxybenzoyl)amino]caprylate, SNAC) were developed to enhance the oral bioavailability of berberine hydrochloride (BER) with poor intestinal membrane permeability. Microspheres were prepared and characterized by particle size measurements, scanning electron microscopy, differential scanning calorimetry, BER payload and release, Caco-2 cell monolayer transport, and rat pharmacokinetics. The microspheres were spherical and had uniform size, high encapsulation efficiency and high loading capacity. In vitro release studies showed that BER-loaded microspheres had good sustained release characteristics. The Caco-2 cell monolayer transport study proved that SNAC could significantly enhance permeability of BER 2–3-fold. Pharmacokinetic studies demonstrated a 9.87-fold increase in area under the curve (AUC) of BER mixed with SNAC and a 14.14-fold increase in AUC of microspheres compared with BER alone. These findings indicate that SNAC is a promising absorption enhancer for oral delivery of BER in the form of both solution and microspheres.

## 1. Introduction

Berberine hydrochloride (BER) is a kind of quaternary ammonium type isoquinoline alkaloid; it is extracted from *Coptis chinensis*, a kind of plant, as both medicine and food. BER shows a curative effect in the medication of various diseases. For example, BER shows a good effect in clinical treatment of type 2 diabetes mellitus and improves glucose and lipid utility [1,2]; provides neuroprotective effects in TgCRND8 mice [3]; prevents kidney damage by protecting the structure and function of the kidney and inhibiting the proliferation and secretion of mesangial cells [4]; ameliorates acute endotoxemia [5]; and shows good effect in treatment of endometritis [6].

BER is easily adsorbed by protein, which makes it difficult to achieve effective concentrations [7,8,9,10,11]. Furthermore, as BER is slightly soluble in water and has poor biological membrane permeability, it shows poor intestinal absorption and very low oral bioavailability [12,13]. The conventional dosage forms of BER for oral administration cannot reach the therapeutic concentration with only a few administration times; it usually takes several months to take therapeutic effect [14]. A high dosage of BER can bring toxicity and side effects to the bodies of patients [15,16]. Obviously, developing an efficient drug delivery system for enhancing oral bioavailability of BER is a potential strategy for extending the clinical application of BER.

Microspheres are small spherical particles, usually with a particle size between 1 and 250 μm. They are produced as a kind of natural or synthetic polymer material encapsulating drugs. As a new drug delivery technology, microsphere technology can achieve the long-term effect of sustained release by adjusting and controlling the release rate of drugs [17,18,19]. At the same time, it can protect drugs from degradation to improve drug stability; improve drug bioavailability to improve efficacy; and reduce drug administration times and drug stimulation to reduce toxicity and side effects [20].

To overcome BER’s poor permeability and adherence to the mucus, the effective and safe absorption enhancer sodium N-[8-(2-hydroxybenzoyl)amino]caprylate (SNAC) is used with BER to enhance its membrane permeation, and they are loaded into microspheres to decrease their adherence to the mucus. A previous study showed that SNAC could significantly increase the oral bioavailability of salvianolic acids and notoginsenoside R1 without any serious mucosal damage [21]. SNAC is widely used in the oral absorption of different kinds of drugs, including proteins and other macromolecules, such as insulin, calcitonin and heparin [22,23,24]. SNAC has been applied to the enhancement of oral bioavailability for long-acting GLP-1 analog (semaglutide) to treat type 2 diabetes [25]. Oral semaglutide approved for oral administration has been granted in the USA by FDA [26,27]. A product that uses SNAC co-administration with vitamin B12 for improving its oral absorption is already on the market [28].

Therefore, this study aimed to develop a microsphere drug delivery system with an absorption enhancer for oral administration of BER, and SNAC was used as the absorption enhancer. The release profiles in vitro, transport across Caco-2 cell monolayer and rat pharmacokinetics of BER were investigated. The drug delivery system may be a potential carrier, providing a higher level of intestinal membrane permeation and sustained release.

## 2. Results and Discussion

### 2.1. Sodium N-[8-(2-hydroxybenzoyl)amino]caprylate Determination

**Figure 1:**
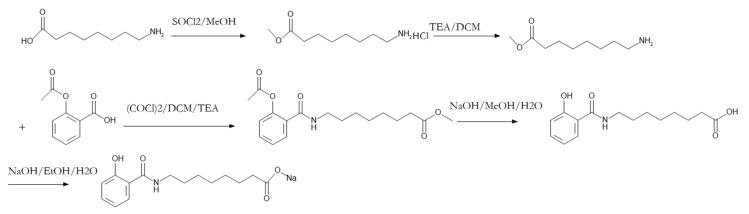
The synthesis process of *N*-[8-(2-hydroxybenzoyl)amino]caprylate (SNAC).

The HPLC chromatogram result demonstrated the purity of SNAC was 99.76%. The LCMS chromatogram result indicated that the purity of SNAC was 98.86%. The synthesis process is shown in Figure 1.

### 2.2. Cytotoxicity Assay by MTT Test

The viability of Caco-2 cells after incubation with BER for 24 h was calculated. The experimental concentration range of drugs that kept more than 90% cells still alive was considered the safe concentration for study. When the concentration of BER was equal or lower than 100 μg·mL^−1^, the cell survival rate was higher than 90%. Therefore, the safe concentration for study of BER was equal or lower than 100 μg·mL^−1^. The safe concentration for study of SNAC was equal or lower than 200 μg·mL^−1^ [21].

### 2.3. Caco-2 Cell Monolayer Transport

**Figure 2:**
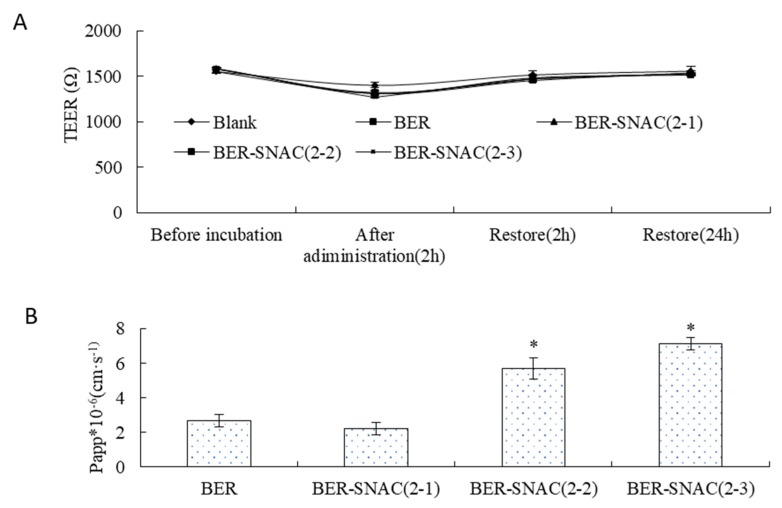
Transport across the Caco-2 cell monolayer. (**A**) Transepithelial electrical resistance (TEER) of Caco-2 cell monolayer before and after treatment with berberine hydrochloride (BER) and BER mixed with sodium *N*-[8-(2-hydroxybenzoyl)amino]caprylate (SNAC). (**B**) Papp of BER transport across Caco-2 cell monolayer treatment with BER and BER mixed with SNAC. Data were represented as mean ± SD (*n* = 3, each group). * *p* < 0.05 vs. BER group.

As shown in Figure 2A, the TEER showed no significant change before and after transport across the Caco-2 cell monolayer, which suggests that BER might not transport across the Caco-2 cell monolayer by the paracellular route. Then, after re-incubation for 2 h and 24 h with complete medium, TEER restored to its original state, which indicates the drugs had no toxicity to cells. Figure 2B displays the Papp values of BER mixed with SNAC at different ratios for transport across the Caco-2 cell monolayer; compared with the Papp of BER solution, the Papp of BER mixed with SNAC (2:1) showed no significant difference, and the Papp of BER mixed with SNAC (1:1) and BER mixed with SNAC (2:3) improved by 2.11-fold and 2.64-fold. SNAC promoted drug transmembrane transport in a dose-dependent manner. The more SNAC was used, the stronger the enhancement effect on BER transport. The Papp of BER mixed with SNAC (1:1) and BER mixed with SNAC (2:3) had no significant difference, which indicates that BER mixed with SNAC (1:1) can be chosen for enhancing the transport of BER. The mechanisms by which absorption enhancers promote oral absorption of drugs mainly include transcellular pathways and paracellular pathways via opening tight junctions of gastrointestinal epithelial cells [29,30]. BER mixed with SNAC did not transport across the Caco-2 cell monolayer by opening tight junctions.

### 2.4. Characterization of Microspheres

There are many methods for preparing microspheres. In this experiment, ethyl cellulose was used as the carrier material and acetone was used as the solvent with suitable polarity. To dissolve the drug in the organic solvent of the carrier material, the organic phase is added to the water phase to emulsify, the water–oil emulsion is obtained, then the emulsion is slowly poured over the water. The organic solvent diffusion extraction, as a result of the solubility of the carrier material reduced, results in the formation of microspheres. The solvent volatilization method is simple and easy to operate, which is also a common method to prepare microspheres.

**Figure 3:**
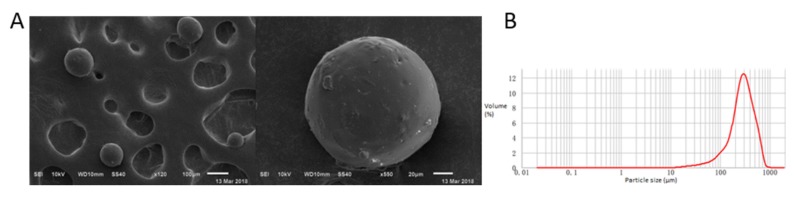
Characterization of microspheres. (**A**) Scanning electron microscope. (**B**) Particle size distribution.

Microspheres’ morphology was investigated (Figure 3A); the particle sizes were measured (Figure 3B). The results showed that the preparation repeatability was good, microspheres was spherical, and the volume median diameter of microsphere particles (D (v, 0.5)) was (275.92 ± 14.02) μm. The LC was 21.90% ± 1.50%. The EE was 65.52% ± 2.45%. The results indicated that microspheres could load a large amount of BER, and BER-loaded microspheres had high EE.

### 2.5. Differential Scanning Calorimetry (DSC) Measurements

**Figure 4:**
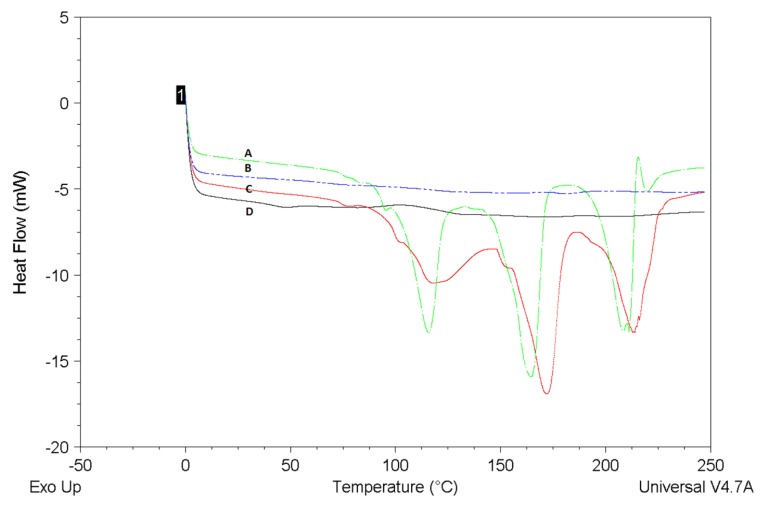
Differential scanning calorimetry (DSC) study. DSC endothermal curves of berberine hydrochloride (BER) (**A**), *N*-[8-(2-hydroxybenzoyl)amino]caprylate (SNAC)-loaded microspheres (**B**), physical mixture of BER/SNAC-loaded microspheres (**C**), and BER-SNAC-loaded microspheres (**D**).

DSC curves of BER-SNAC-loaded microspheres were drawn in comparison with SNAC-loaded microspheres, BER, and their physical mixture (BER/SNAC-loaded microspheres MIX) Figure 4. The DSC endotherm of the BER sample showed three endothermal peaks. The DSC endotherm of the SNAC-loaded microspheres sample showed no melting endotherm. BER/SNAC-loaded microspheres MIX also showed the same three endothermal peaks as BER, which were the endothermal peaks of BER. However, the typical endothermic peaks of BER were not observed in the curve of the BER-SNAC-loaded microspheres, which illustrates the molecular encapsulation of BER inside the microspheres and the high EE of BER.

### 2.6. In Vitro Release

**Figure 5:**
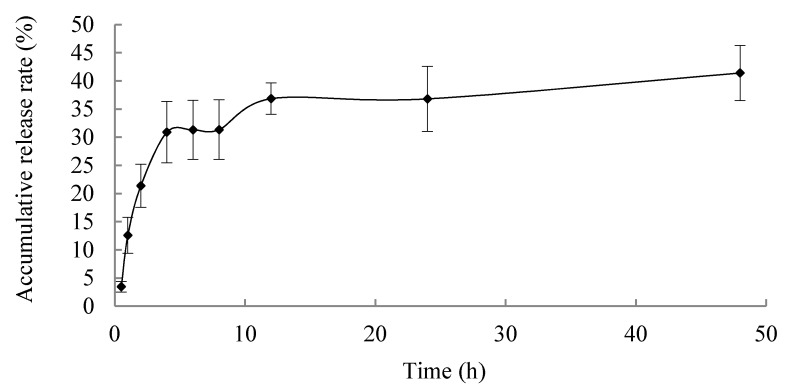
In vitro release study of berberine hydrochloride-loaded microspheres. Data are represented as mean ± SD (*n* = 3, each group).

In vitro release profile is shown in Figure 5; the microspheres exhibited a sustained release properties, and the accumulative release rate of BER for 48 h was less than 40%, which is promising for reducing the frequency of drug administration, improving the therapeutic efficacy and reducing side effects caused by frequent administration of BER.

### 2.7. Berberine Hydrochloride (BER) Chromatogram and Calibration Curve

**Figure 6:**
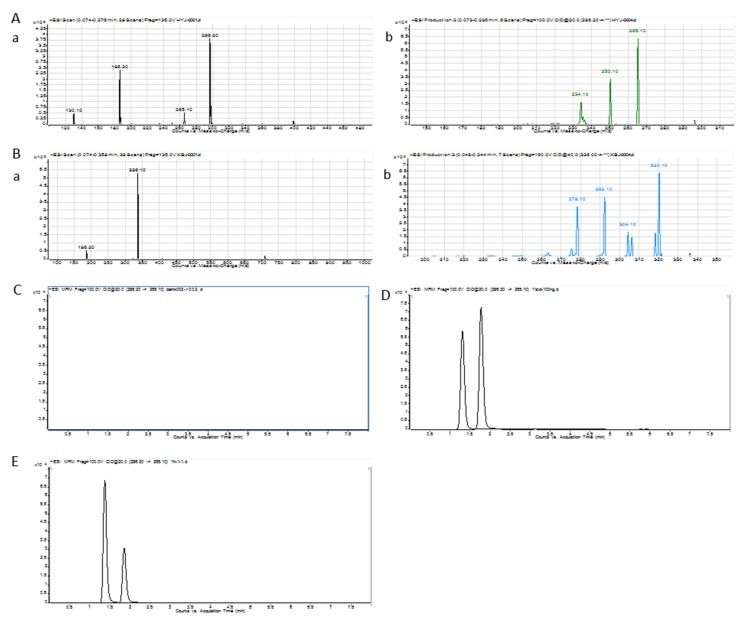
UPLC/MS/MS ion chromatogram of nuciferine (**A**) a.) Total ion chromatogram b.) Product ion chromatogram. UPLC/MS/MS ion chromatogram of berberine hydrochloride (BER) (**B**) a.) Total ion chromatogram b.) Product ion chromatogram. Rat blank plasma (**C**). Rat plasma was added with the corresponding standard. (**D**) Blood sample with administration of BER (**E**), 1. nuciferine; 2. BER.

The UPLC/MS/MS ion chromatogram condition was investigated to get a high separation and sensitivity peak [31]. The UPLC/MS/MS ion chromatogram of BER is shown in Figure 6A,B. The total ion chromatogram and product ion chromatogram of BER were 336.1 and 292.1, respectively. The total ion chromatogram and product ion chromatogram of nuciferine were 296.2 and 265.1, respectively. Figure 6C–E display the chromatogram of blank blood, blank-blood-spiked BER and nuciferine, and processed sample after the administration of BER, respectively. The peaks of nuciferine and BER could be separated well. There was no peak in blank blood at the same retention time. 

### 2.8. Method Validation

Calibration curves of BER showed that good linearity was achieved over a range of concentrations from 1.0 ng·mL^−1^ to 100.0 ng·mL^−1^ for BER (correlation coefficient > 0.98). The standard curve equation was y = 0.5313x − 0.0020; y represents peak area of BER/peak area of nuciferine, x represents concentration of BER/concentration of nuciferine.

The result for precision was less than 10%, which met the required criteria. The results for recovery were found in a range from 85% to 115%, which met analytical requirements. The matrix effect was observed and was in the acceptable range from 95% to 105%. The LOQ that could be detected in plasma was 0.1 ng·mL^−1^, which displayed that instrument detection sensitivity was high enough.

### 2.9. Pharmacokinetics

**Figure 7:**
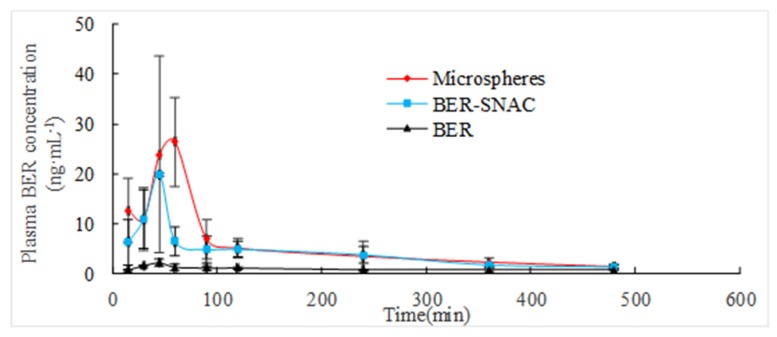
Pharmacokinetic profile for total berberine hydrochloride (BER) content vs. time in rats after oral administration of BER, BER mixed with sodium *N*-[8-(2-hydroxybenzoyl)amino]caprylate (SNAC), and BER-SNAC-loaded microspheres. Means ± S.D., *n* = 6.

The concentration–time curve of BER in plasma is shown in Figure 7. It was evident that area under the curve (AUC) of BER after administration of BER mixed with SNAC was 9.87-times higher than after administration of BER. Moreover, AUC of BER after administration of BER/SNAC-loaded microspheres was 14.14-times higher than after administration of BER. The results displayed significant absorption enhancement after administration of BER mixed with SNAC. T_max_ of BER after administration of BER mixed with SNAC was significantly larger than after administration of BER (*p* < 0.05), which displayed significant slow release after administration of BER mixed with SNAC. The possible mechanism was that BER may produce dipole–dipole non-covalent interactions with SNAC, which caused conformational change to expose hydrophobic regions. This interaction complex could easily permeate gastrointestinal epithelial cell membranes. The complex then dissociated into BER and SNAC [21]. Thereby, SNAC could promote ionized molecules’ membrane permeability and could prolong retention times in blood. Tmax of BER/SNAC-loaded microspheres in blood was prolonged, with significant differences compared to BER mixed with SNAC and BER (*p* < 0.05), which showed that microspheres had sustained release properties.

In this study, BER/SNAC-loaded microspheres were developed to enhance the oral bioavailability of BER. SNAC may promote permeation through the gastrointestinal epithelial cell membrane of BER via a transcellular pathway, which significantly improves the absorption of BER and enhances oral bioavailability. Microspheres could prolong the retention time of BER in blood and improve the oral bioavailability of BER more substantially.

## 3. Materials and Methods

### 3.1. Materials

Berberine hydrochloride and Nuciferine were both supplied by the National Institute of the Control of Pharmaceutical and Biological Products (Beijing, China). *N*-[8-(2-Hydroxybenzoyl)amino]caprylate (SNAC) was provided by Shanghai Synmedia Chemical Co., Ltd. (Shanghai, China). Ethyl cellulose was provided by Aladdin Industrial Corporation (Shanghai, China).

Specific pathogen-free grade Sprague–Dawley male rats with body mass (250 ± 20 g) were bought from Vital River Laboratories, Beijing, China. Rats were maintained in accordance with the Guide for the Care and Use of Laboratory Animals, and the study was approved by the Ethical Committee of the Experimental Animal Center of the Institute of Medicinal Plant Development, Chinese Academy of Medical Sciences and Peking Union Medical College.

### 3.2. SNAC Synthesis and Determination

The synthesis process of SNAC followed a previous report [21]; 8-aminooctanoic acid was dissolved in methanol containing thionyl chloride, stirred overnight. Then, triethylamine (TEA) dissolved into dichloromethane (DCM) was added, then acetylsalicylate dissolved into DCM containing oxalylchloride and TEA was added. The above products were dissolved into methanol and added to sodium hydroxide solution. The ester was hydrolyzed to get the carboxyl acid. Then it was dissolved into ethanol and sodium hydroxide solution was accurately added to produce the sodium salt. The purity of SNAC was analyzed with HPLC-UV (Agilent 1260) and HPLC-MS/MS (Agilent 1200-6130). 

### 3.3. MTT Study

The Caco-2 cells concentration was adjusted to 5 × 10^4^ /mL and cultured in a 96-well plate with 200 μL/ hole for 24 h. The experiment was conducted when the confluence of cells reached 80%. BER of different concentrations was diluted in the medium (8 concentration gradients, six replicates for each concentration) 200 μL was added in each hole. The control group (medium containing 10% FBS serum and cells) and the blank group (medium containing 10% FBS serum) were set. They were incubated for 24 h, washed twice with d-hanks solution, and added to 200 μL fresh medium and 20 μL MTT solution (0.5 mg·mL^−1^), incubating for 4 h. They were washed twice with d-hanks solution, added to with 150 μL DMSO, and oscillated on a plate oscillator for 10 min. The optical density (OD) value of each hole was measured at wavelength 570 nm by MQX200 microplate reader (Bio-Tek, USA). The formula of relative cell survival rate was as follows:

Relative cell survival rate (%) = OD of the drugs group/OD of the control group × 100%

### 3.4. Caco-2 Cell Monolayer Transport

Caco-2 cells were inoculated into Transwell plate at a concentration of 2.0 × 10^5^ /mL for conventional culture. After incubation for 21 days, they were washed three times with D-Hanks. The apical (AP) side was added to with a certain concentration of 0.5 mL BER, mixed with SNAC (2:1, 1:1, 2:3) (D-Hank’s solution, pH = 7.4), and the basolateral (BL) side was added to with 1.5 mL D-Hanks, then incubated in at 37 °C water bath oscillator for 2 h. Trans epithellal electric resistance (TEER) was determined using a Millicell-ER system (Millipore Corporation, Bedford, MA, USA) before and after the transport experiment. In addition, after the transport experiment, the cells were washed 3 times with d-hanks and cultured in complete medium for 2 h and 24 h. TEER values were determined again to investigate the toxicity of drugs to cells and cell membrane regeneration. The sample from BL was injected into the HPLC system for measuring BER content. The formula of apparent permeability coeffcient (Papp) was as follows:

Papp= dQ/dt × 1/(AC_0_), where dQ/dt is the permeability rate, C_0_ is the initial concentration at the apical (AP) side, and A is the surface area of a monolayer.

### 3.5. Microspheres Preparation

BER and SNAC (ratio = 1:1) was evenly dispersed in an acetone solution of ethyl cellulose in a beaker. Under the condition of room temperature and mechanical stirring (the stirring speed is 800 rpm), the solution was slowly added into liquid paraffin with sorbitan oleate (span 80) as an emulsifier. After emulsification for a certain time, acetone was removed by evaporation. After the acetone was exhausted, the microspheres were filtered and collected. The microspheres were washed with petroleum ether and dried in vacuum at room temperature.

### 3.6. Loading Capacity and Encapsulation Efficiency

The microspheres were washed 3 times with 95% methanol, and the washing liquid was discarded. The microsphere was ground 3 times with 95% methanol. The supernatant was filtered, and the content of BER encapsulated in microspheres was determined by HPLC-UV. The loading capacity (LC) and encapsulation efficiency (EE) of BER were subsequently determined using Equations (1) and (2), respectively.

LC (%) = Mass of BER in microsphere/total mass of microsphere × 100%(1)

EE (%) = Mass of BER in microsphere/total mass of input BER × 100%(2)

### 3.7. Microsphere Characterization

Size was determined by a Mastersizer 2000 particle analyzer (Malvern Instruments Ltd., Worcestershire, UK). Appearance was determined by scanning electron microscope (SEM) (JSM-6510LV, JEOL, Tokyo, JAPAN). The microspheres were analyzed by differential scanning calorimetery (DSC) evaluation (Q200, TA Instruments, New Castle, DE, USA), with a heating rate of 10 °C·min^−1^ from 0 to 250 °C.

### 3.8. In Vitro Release

Microspheres of BER were placed in a dialysis bag with in particular amounts, and phosphate buffer solution (PBS, PH 7.4) was added to the dialysis bag. Then, the dialysis bag was placed in 50 mL PBS and placed over an orbital shaking bath (SHA-B (A), Jintankexi apparatus Co. Ltd., Jiangsu, China). The rotation speed was 100 rpm. The temperature was 37 °C. A quantity of 1.0 mL of sample solution was withdrawn from the medium at 0.5, 1, 2, 4, 6, 8, 12, 24, 48 h; then, the sample was filtered and determined by HPLC-UV. Equal volume of fresh medium was replaced. The measurement was carried out three times, and the cumulative release % of BER from microspheres was calculated as the following formula:

Cumulative release % = amount of BER released/amount of initial BER × 100%

### 3.9. Pharmacokinetics Sample Analysis

#### 3.9.1. Chromatographic System and Conditions

The analytical column C18 was Phenomenex Kinetex^®^ EVO C18 (2.1 × 50 mm; 2.6 μm). The mobile phase consisted of acetonitrile (containing 0.1% formic acid) (A) and water (containing 0.1% formic acid) (B). The flow rate was 0.3 mL·min^−1^. The column temperature was 25 °C. The elution was carried out as follows: 20% A at 0−2.0 min; 20–100% A at 2.0−4.0 min; 100% A at 4.0−5.0 min; 100–20% A at 5.0−5.1 min; 20% A at 5.1−8.0 min. The injection volume was 10 μL. The mass spectrometer operation was in positive ionization mode to assess the BER and nuciferine: *m/z* 336.1/292.1 for BER, *m/z* 296.2/265.1 for nuciferine (interior label, IS). The optimized fragmentor and collision energy were 160 V and 40 eV for BER, 100 V and 20 eV for nuciferine, respectively. Gas temp. was 325 °C. Nebulizer was 45 psi. Gas flow was 8 L·min^−1^. Capillary was 4000 V. Nozzle voltage was 1000 V. Data acquisition and processing were accomplished on a 1290/6460 triple quadrupole mass spectrometer (Agilent Technologies Inc., CA, USA).

#### 3.9.2. Preparation of Serum Samples

The 100 μL of serum sample with nuciferine was added to 790 μL acetonitrile, whirled on a vortex for 5 min and centrifuged at 14,000 rpm for 10 min. The supernatant was collected and transferred to a clean centrifuge tube. The sediment was whirled on a vortex with 800 μL acetonitrile for 5 min and centrifuged at 14,000 rpm for 10 min. The supernatant was transferred to the first supernatant. The solution was evaporated at room temperature to dryness by a vacuum centrifugal thickener (Centrivap, LABCONCO, Kansas, MO, USA). The residue was dissolved in 100 μL of 20% aqueous acetonitrile solution, whirled on a vortex for 3 min and centrifuged at 14,000 rpm for 10 min for twice, and determined using the condition as 3.9.1.

#### 3.9.3. Method Validation

The selectivity, linearity, method recovery, precision, matrix effects, and lower limit of quantitation (LOQ) were determined for the validation of the bio-analytical method.

The blank serum, blank serum spiked with BER, and serum samples after administration of BER were taken, and the samples were processed according to the method “3.9.2”. The chromatograms of the three samples were compared to assess the specificity of the method.

The blank serum was obtained from the blank rats and added with different concentrations of BER standard solutions, whirling and adding the appropriate amount of nuciferine standard solution. The samples were processed as “3.9.2.”. Linear regression was conducted based on the abscissa of the ratio of BER content and IS content and the ordinate of the ratio of BER peak area and IS peak area.

BER standard solutions was taken in centrifugal tube. A quantity of 100 μL of blank serum was added to obtain serum with a concentration of 3, 30 and 80 ng·mL^−1^. Samples were processed according to method “3.9.2” and analysis. BER content was calculated according to the standard curve. The content values were compared with the theoretical values to calculate the method recovery.

Serum with 3, 30 and 80 ng·mL^−1^ of BER was processed according to method “3.9.2” and analyzed in six replicates with the same analytical run to assess the precision.

Blank serum spiked with 3, 30 and 80 ng·mL^−1^ of BER standard sample was taken and processed according to method “3.9.2”. Acetonitrile spiked with 3, 30 and 80 ng·mL^−1^ of BER standard sample was also processed with the same method. Then, the two sets of samples were compared. The ratio was calculated to assess the matrix effect.

The LOQ of BER under the chromatographic conditions was determined at a signal/noise value of about 10.

### 3.10. Administration of BER

Male sprague dawley (SD) rats with a weight range of 250 ± 20 g were used as the animal model. The rats were divided into three groups, 6 rats in each group. The rats were orally administered with the equivalent 200 mg/kg of BER (BER solution group, BER mixed with SNAC group, BER/SNAC-loaded microspheres). A quantity of 500 μL of the blood of the rats in each group was withdrawn from the rat orbital vein at specific time-points. The blood samples were immediately centrifuged at 1000 rpm for 10 min and 4 °C, and the supernatant plasma was transferred to a new centrifuge tube. The serum samples were processed with method “3.9.2” and stored at 20 °C for analysis.

### 3.11. Data Processing

A Phoenix WinNonlin 6.0 (Pharsight, CA, USA) was used to calculate pharmacokinetics parameters. The statistical method of two-way ANOVA was used to judge data statistical differences by the SPSS16.0 Software. The *p* < 0.05 level was considered as statistical significance.

## 4. Conclusions

In this study, microspheres containing SNAC were used to enhance the oral bioavailability of BER. SNAC was synthesized with a purity of about 99%. A caco-2 cell transport experiment was conducted to preliminarily investigate the mechanism of SNAC to enhance the absorption of BER via the gastrointestinal tract. SNAC promoted the transmembrane absorption of BER, which was not a mechanism regulating tight junction permeability. BER-loaded microspheres containing SNAC were developed, and the particle size and electron microscopy of the microspheres were investigated and characterized. The results showed that the microspheres were spherical with high entrapment efficiency. DSC results showed that BER was encapsulated in microspheres. Pharmacokinetics was conducted to investigate the oral absorption of BER, BER mixed with SNAC and BER-SNAC-loaded microspheres; the results indicated that SNAC promoted the oral absorption of BER; microspheres had a certain sustained release effect, and the AUC was further improved over that of BER mixed with SNAC.
